# Supplementary material for: Multi-Functional MPT Protein as a Therapeutic Agent against Mycobacterium tuberculosis
Source: Biomedicines. 2021 May 13;9(5):545. doi: 10.3390/biomedicines9050545 (PMC8152475; doi:10.3390/biomedicines9050545)
Supplement: Supplementary file 1 [file biomedicines-09-00545-s001.zip › biomedicines-1165288-supplementary.pdf]

Supplemental Information

Supplementary Figure and Legends

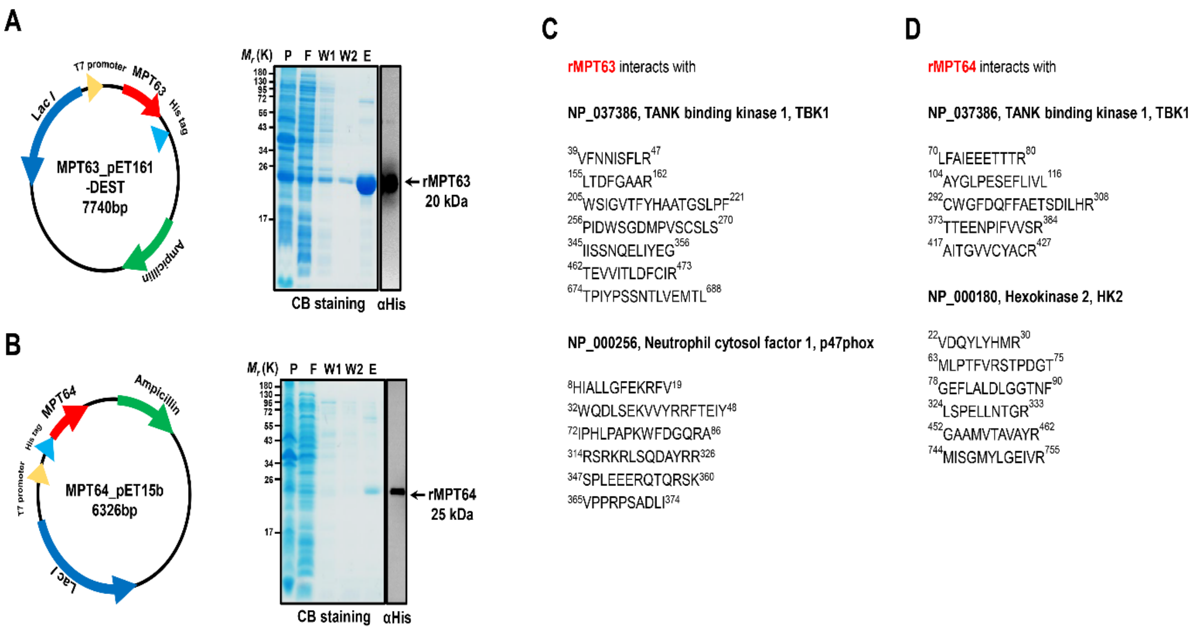

Figure S1. rMPT63 and rMPT63 protein and identified of interacting protein. Construction of plasmid encoding rMPT63 (**A**) or rMPT64 (**B**) with 6X His tag in pET-161-DEST vector or pET-15b vector, respectively (left). Purified protein was analyzed by Coomassie Blue staining (middle) and immunoblot with an anti-His antibody (right). (**C** and **D**) Identified of peptides by mass spectrometry analysis (related to **Fig. 1A** and **Fig. 2A**).

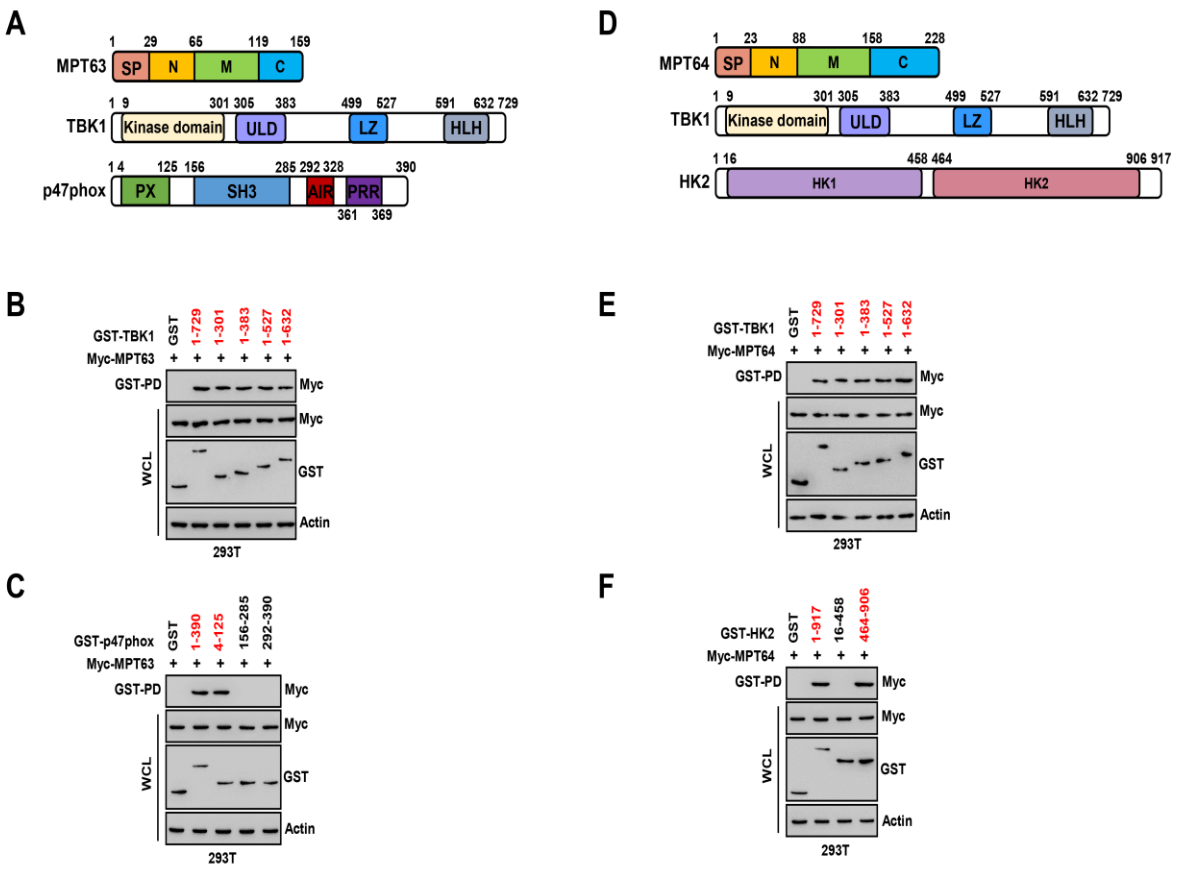

Figure S2. rMPT63 and rMPT63 protein and identified of interacting protein. (**A**) Schematic diagrams of the structure of MPT63, TBK1 or p47phox. (**D**) Structure of MPT6, TBK1 or HK2. 293T cells were co-transfected with mammalian Myc or Myc-MPT63 and truncated mutant constructs together with GST, GST-TBK1 (**B**) or GST-p47phox (**C**), and Myc or Myc-MPT64 and truncated mutant constructs together with GST, GST-TBK1 (**E**) or GST-HK2 (**F**) for 48 hr. 293T cells were used for GST pulldown, followed by IB with αMyc. WCLs were used for IB with αGST, αMyc or αActin (related to **Fig. 1E-H** and **Fig. 2E-H**). The data are representative of four independent experiments with similar results (**B**, **C**, **E**, **F**).

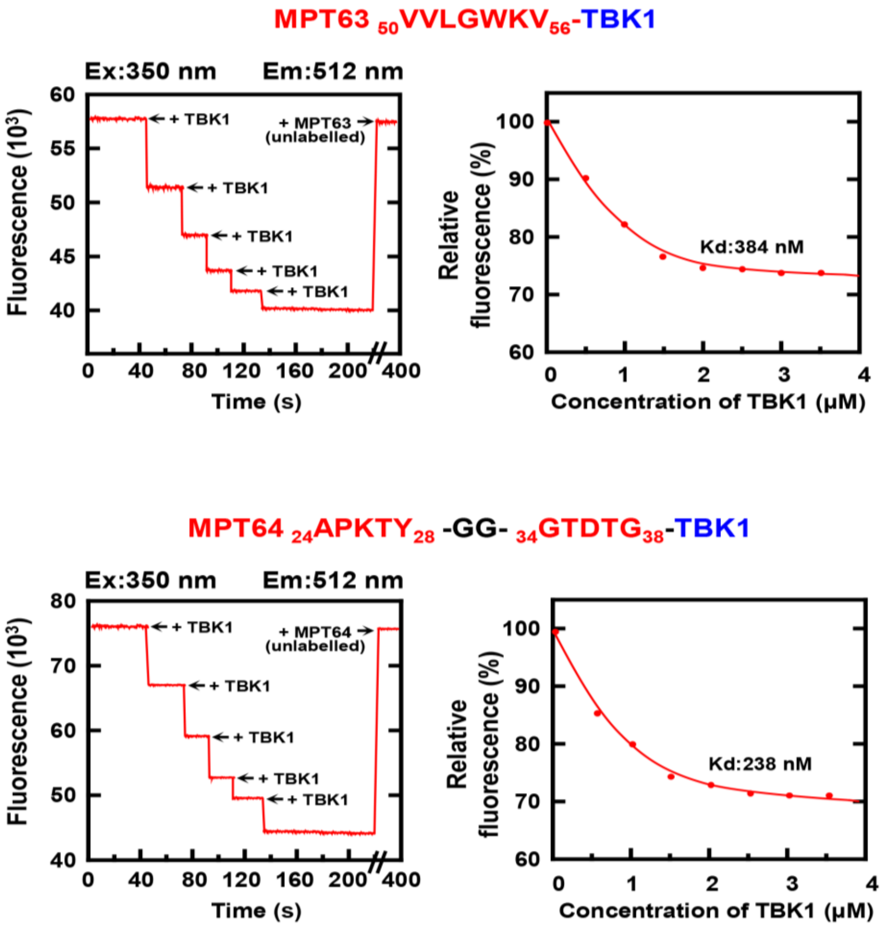

Figure S3. Binding activity of TBK1-MPT63 peptide and TBK1-MPT64 peptide. Titration of fluorescently labelled TBK1 with MPT63 peptide (upper) or MPT64 peptide (bottom), with Kd (384 and 238 nM) determined by curve fit analysis (right) (related to **Fig. 3A**). The data are representative of four independent experiments with similar results.

A

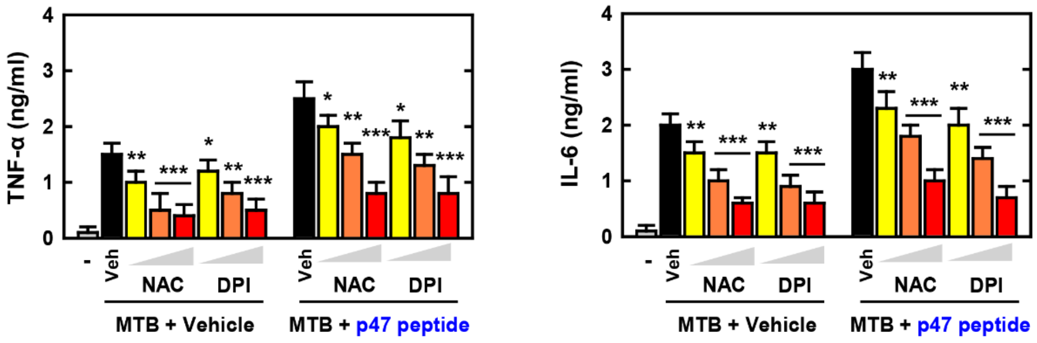

B

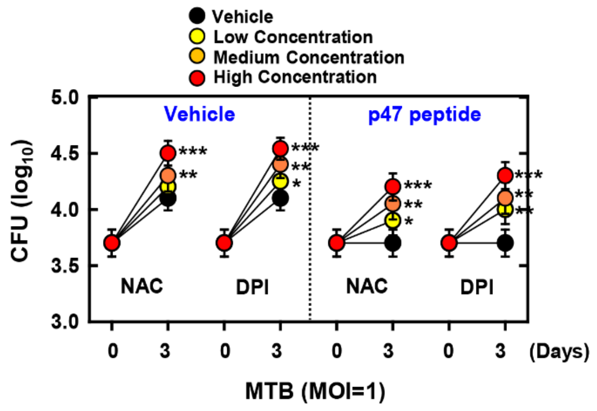

Figure S4. The effect of p47 peptide on proinflammatory cytokine production and antimicrobial activity. (**A**) BMDMs were infected MTB (MOI:1) for 4h and treated with NAC (10, 20, 30 mM) or DPI (5, 10, 20 μM) for 45 min. before Vehicle or p47 peptide (1 μM) for 18 h. The supernatant of BMDMs were used for ELISA to measure the level of TNF-α and IL-6. (**B**) The burdens of MTB in p47 peptide treated-BMDMs were measured after 3 d. The data are representative of four independent experiments with similar results (related to **Fig. 4E and 4F**). Significant differences (*P<0.05; **P<0.01; ***P<0.001) compared with Vehicle-treated BMDMs.

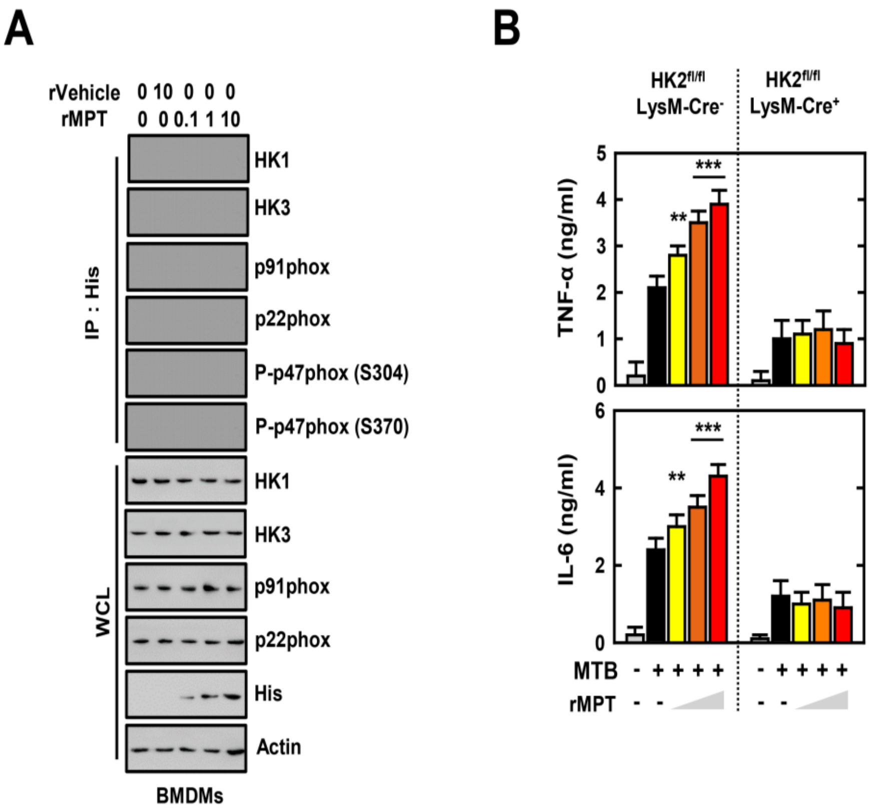

Figure S5. Effects of rMPT-induced inflammation dependent on HK2. (**A**) BMDMs were treated with rMPT (0.1, 1, 10 μg/ml) for 1 hr. Followed by IP with αHis and immunoblotted with αHK1, αHK3, αgp91phox, αp22phox, αp47phox (S304), or αp47phox (S370) and WCL with αHK1, αHK3, αgp91phox, αp22phox, or αActin. The data are representative of four independent experiments with similar results (related to **Fig. 6E**). (**B**) BMDMs from $HK2^{fl/fl}$-LysM-Cre^- and $HK2^{fl/fl}$-LysM-Cre^+ were infected with MTB (MOI = 1) for 4 h and then stimulated with rMPT for 18 h. Culture supernatants were harvested and analyzed for cytokine ELISA for TNF-α and IL-6. Data shown are the means ± SD of five experiments (related to **Fig. 6H**). Significant differences (**$P < 0.01$; ***$P < 0.001$) compared with MTB only.

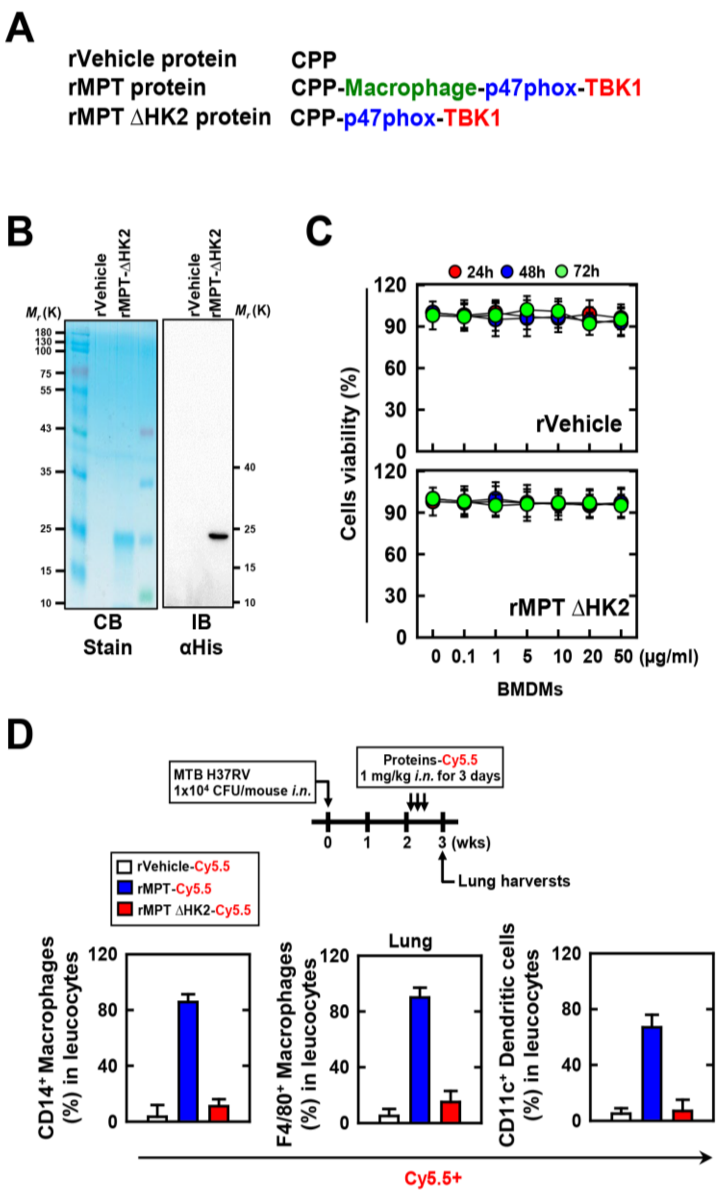

Figure S6. Effects of HK2 of rMPT on mycobacteria-infected macrophages targeting. (**A**) Schematic in design of rMPT and rMPT ΔHK2. (**B**) Bacterially purified 6xHis-rMPT ΔHK2 and rVehicle were analyzed by coomassie blue staining (left) or immunoblotting (IB) with αHis (right). (**C**) BMDMs were incubated with rVehicle or rMPT ΔHK2 for the indicated times and concentration then cell viability measured with MTT assay. (**D**) Mice was infected by MTB through intranasal infection (1 x 10^4/per mice) and intranasally treated Cy5.5-labelled rVehicle, rMPT or rMPT ΔHK2 proteins (1 mg kg-1) after 2 wks. The lung harvests were used for analysis of the numbers of Cy5.5+ cell by FACS. The data are representative of four independent experiments with similar results (**B-D**, related to **Fig. 6**).

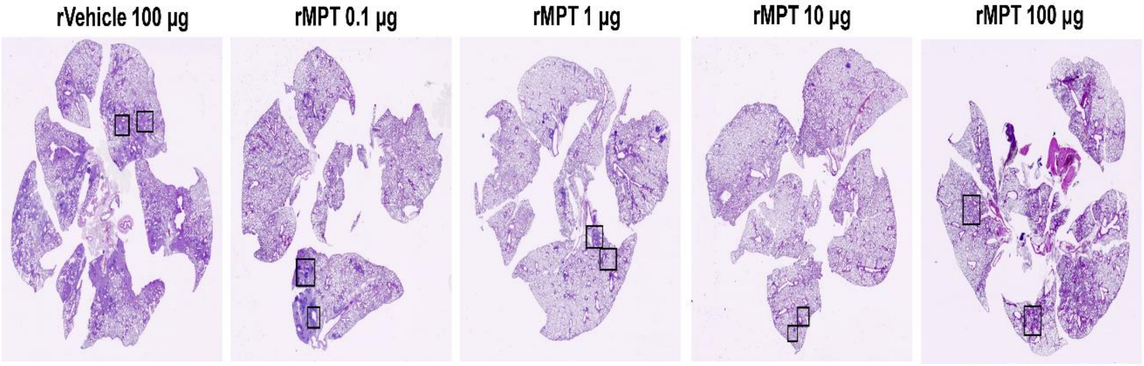

Figure S7. Whole lung photo from H&E stained lung section. The data are representative of ten independent experiments with similar results (related to **Fig. 8B**).

Figure S8. Full-length images of the blots presented in the Figures.

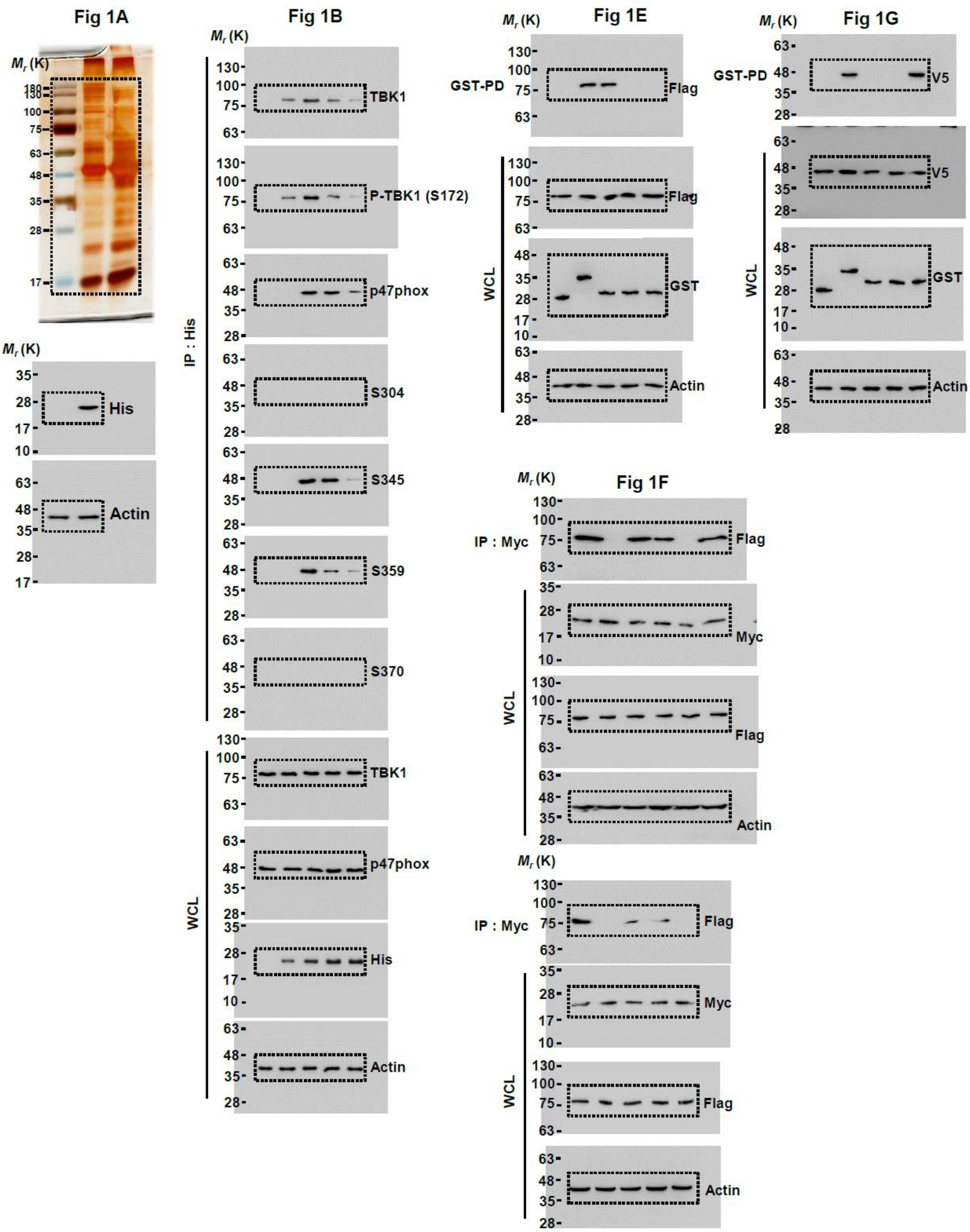

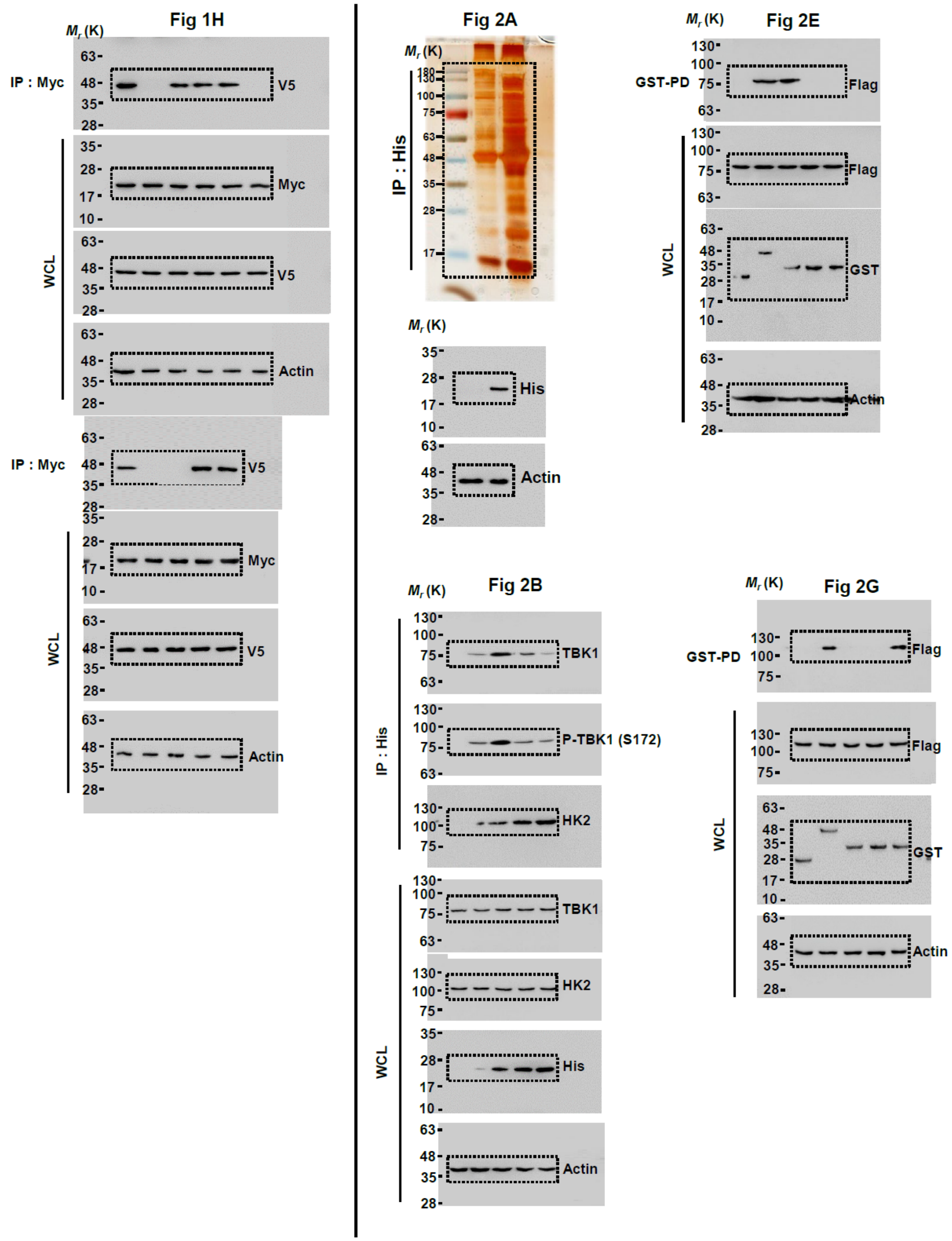
Fig 1H
Mr (K)
IP : Myc
WCL
V5
Myc
Actin
Fig 2A
IP : His
His
Actin
Fig 2B
TBK1
P-TBK1 (S172)
HK2
His
Fig 2E
GST-PD
Flag
GST
Fig 2G

Fig 2F

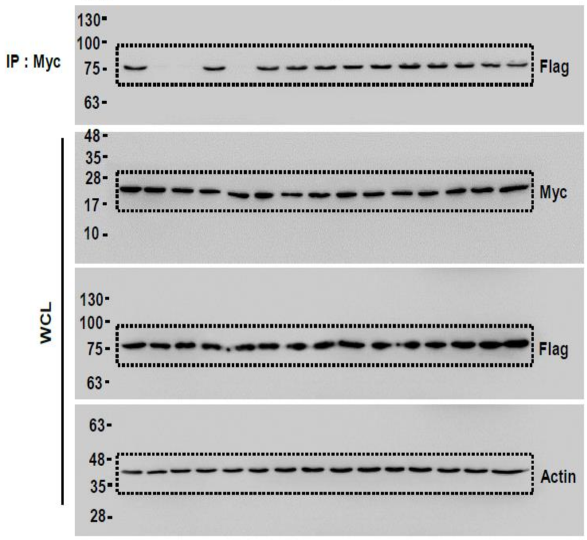

Fig 2H

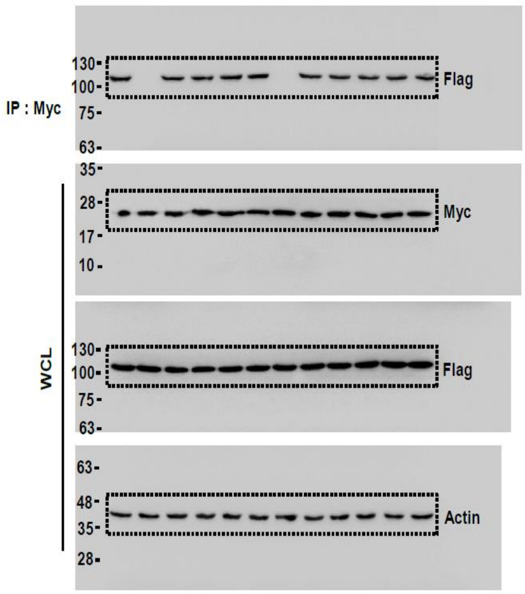

Fig 8D

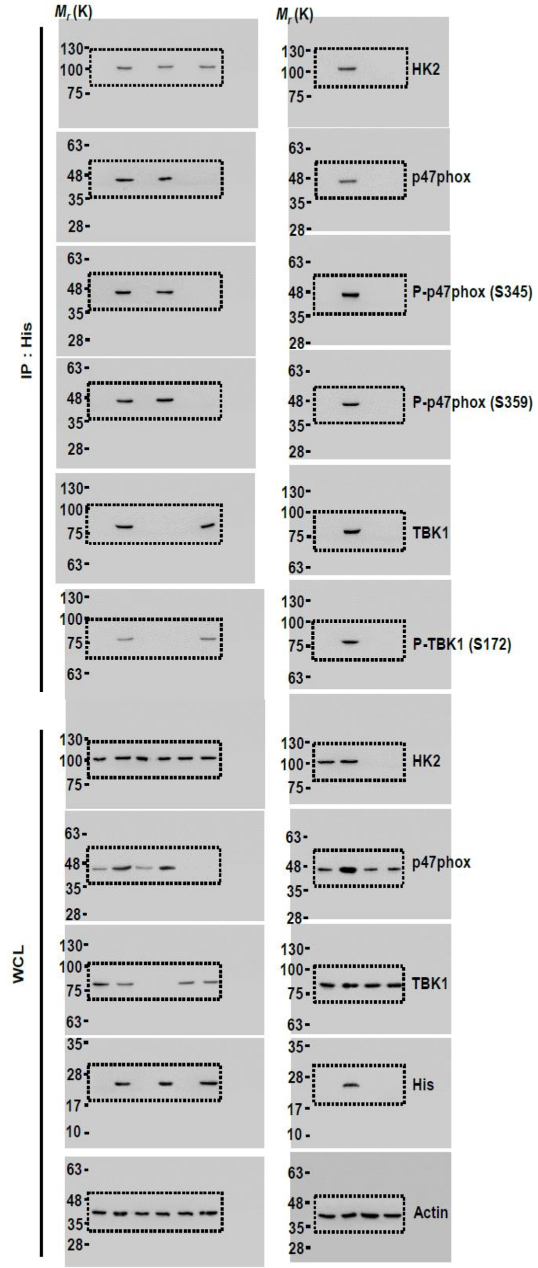

Fig 3B

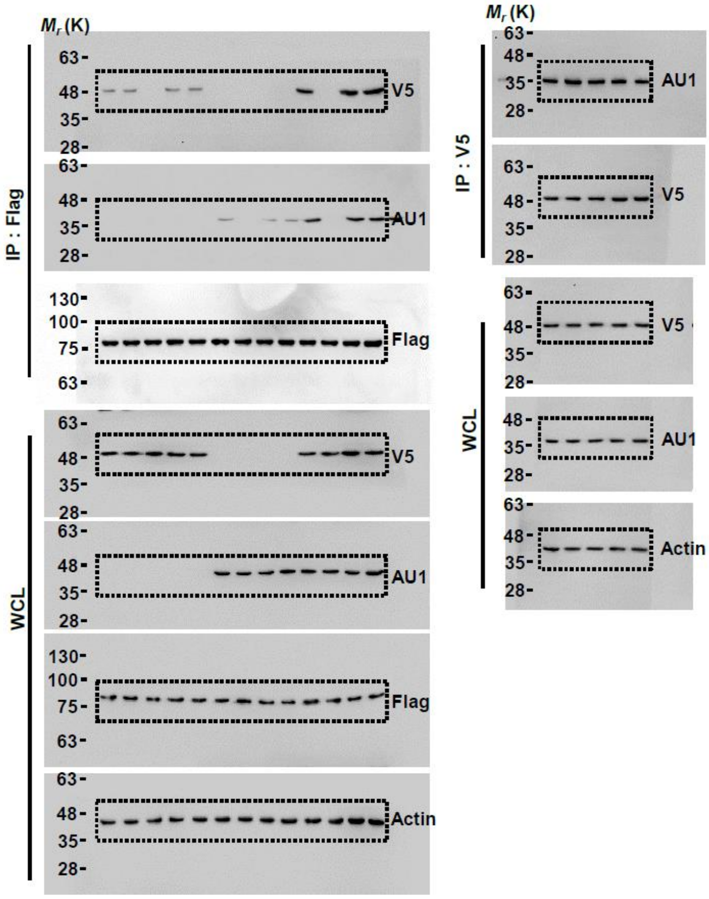

Fig 3C

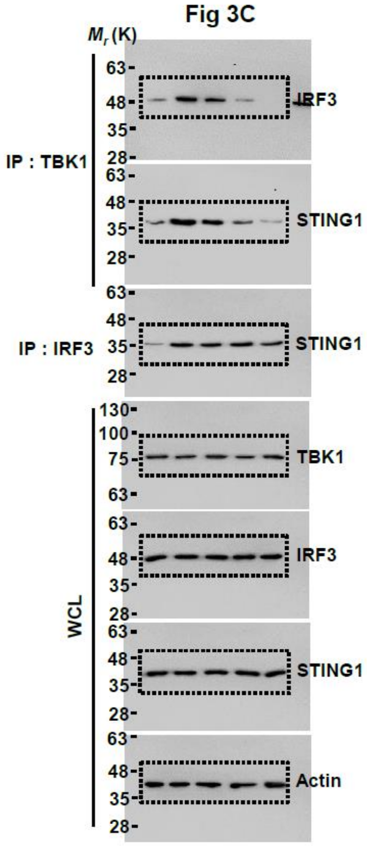

Fig 3D

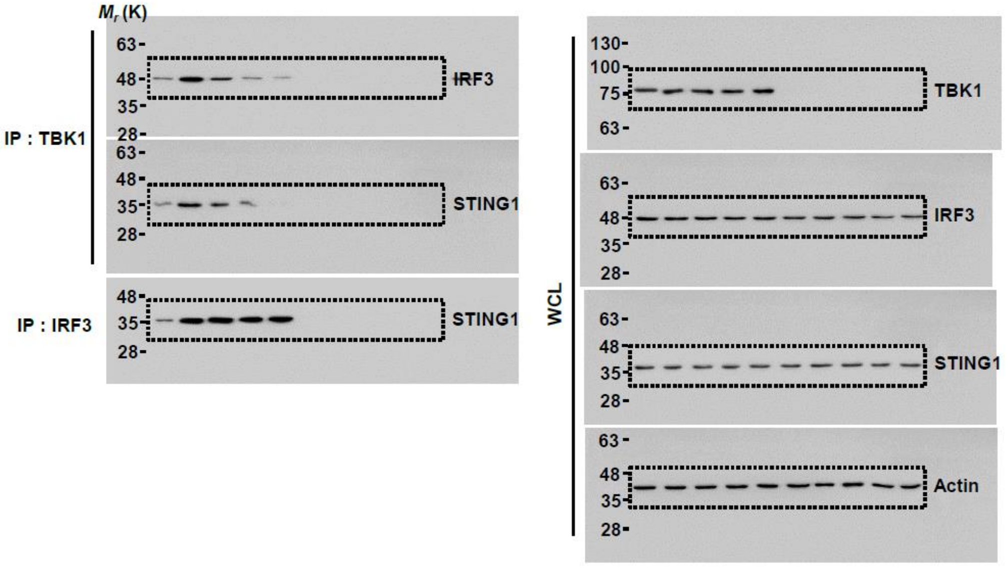

Fig 4A

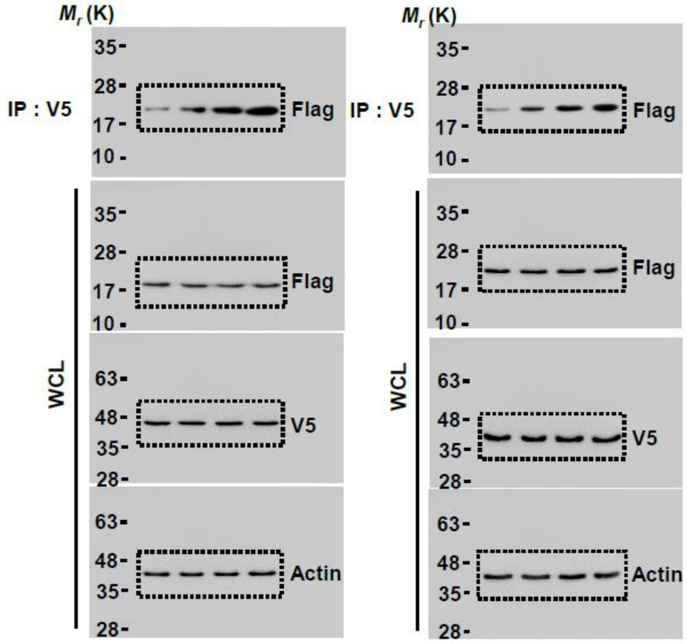

Fig 4B

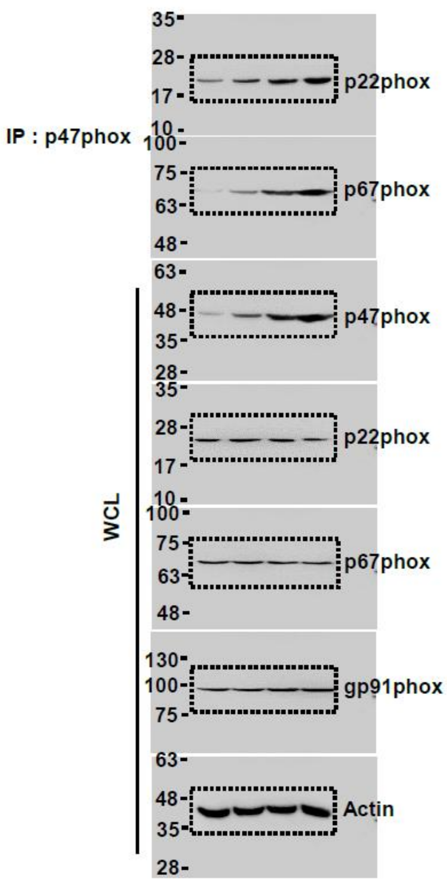

Fig 4C

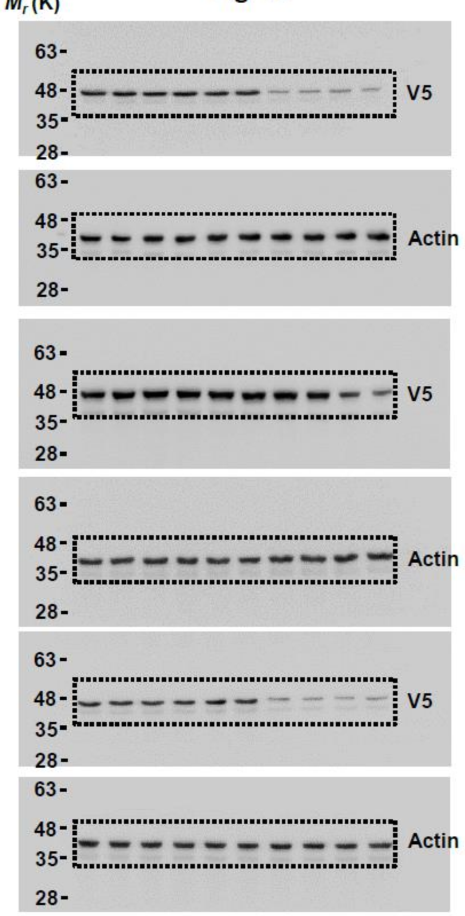

Fig 5A

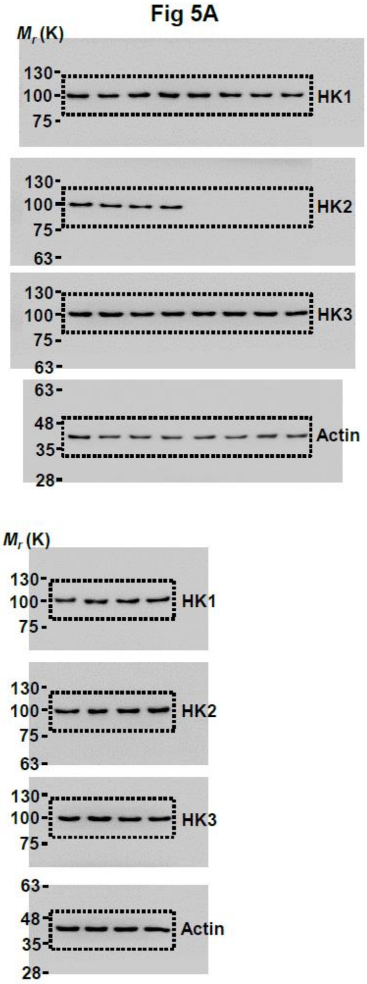

Fig 5B

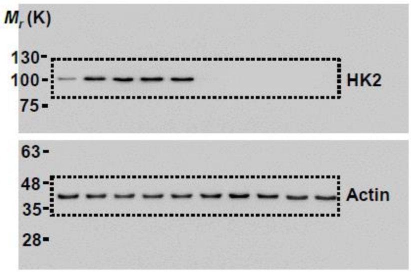

Fig 6E

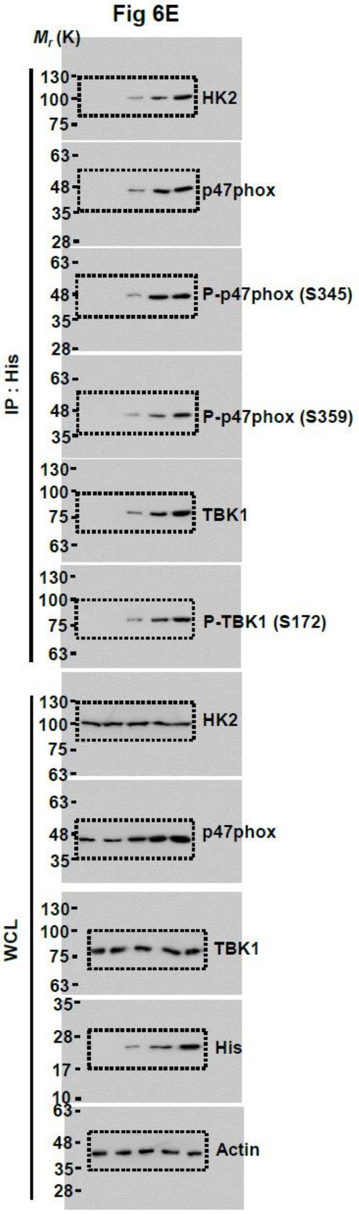

Fig 6F

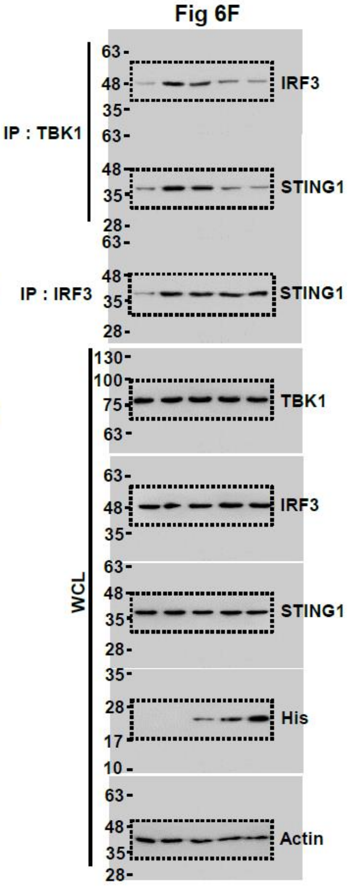

Fig 6G

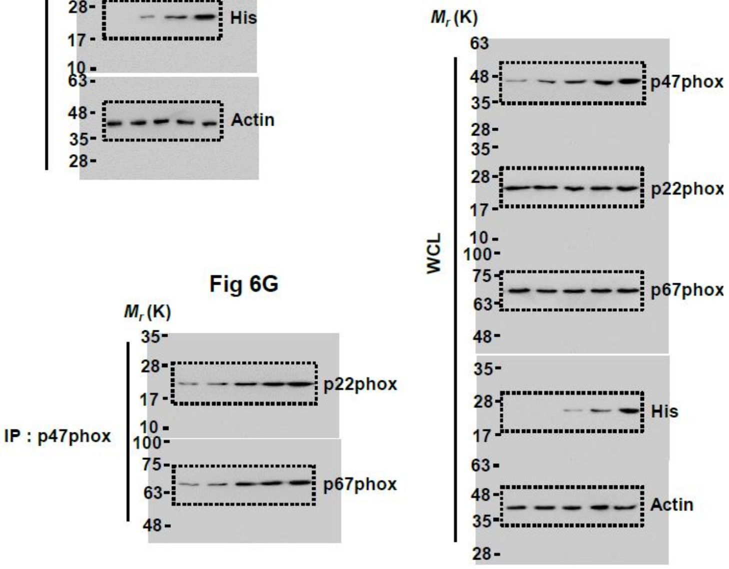

Supporting Experimental Procedures

Protein purification and Mass spectrometry

To identify GRA9-binding proteins, THP-1 cells expressing Flag-GRA9 or vector were harvested and lysed with NP-40 buffer (50 mM HEPES, pH 7.4,150 mM NaCl, 1 mM EDTA, 1% (v/v) NP40) supplemented with a complete protease inhibitor cocktail (Roche, Basel, Switzerland). Post-centrifuged supernatants were precleared with protein A/G beads at 4 °C for 2 h. Precleared lysates were mixed with αFlag antibody-conjugated with agarose beads for 4 h at 4 °C. Precipitates were washed extensively with lysis buffer. Proteins bound to beads were eluted and separated on a Nupage 4-12% Bis-Tris gradient gel (Invitrogen). After silver staining (Invitrogen), specific protein bands were excised and analyzed by ion-trap mass spectrometry at the Korea Basic Science Institute (Seoul, Korea) Mass Spectrometry facility, and amino acid sequences were determined by tandem mass spectrometry and database searches.

GST pulldown, immunoblot, and immunoprecipitation analysis

For GST pulldown, cells were harvested and lysed in NP-40 buffer supplemented with a complete protease inhibitor cocktail (Roche). After centrifugation, the supernatants were precleared with protein A/G beads at 4 °C for 2 h. Pre-cleared lysates were mixed with a 50% slurry of glutathione-conjugated Sepharose beads (Amersham Biosciences), and the binding reaction was incubated for 4 h at 4 °C. Precipitates were washed extensively with lysis buffer. Proteins bound to glutathione beads were eluted with SDS loading buffer by boiling for 5 min.

For immunoprecipitation, cells were harvested and then lysed in NP-40 buffer supplemented with a complete protease inhibitor cocktail (Roche). After pre-clearing with protein A/G agarose beads for 1 h at 4 ˚C, whole-cell lysates were used for immunoprecipitation with the indicated antibodies. Generally, 1-4 µg of commercial antibody was added to 1 ml of cell lysates and incubated at 4°C for 8 to 12 h. After the addition of proteins A/G agarose beads for 6 h, immunoprecipitates were extensively washed with lysis buffer and eluted with SDS loading buffer by boiling for 5 min.

For immunoblotting, polypeptides were resolved by SDS-polyacrylamide gel electrophoresis (PAGE) and transferred to a PVDF membrane (Bio-Rad). Immuno detection was achieved with specific antibodies. Antibody binding was visualized by chemiluminescence (ECL; Millipore) and detected by a Vilber chemiluminescence analyzer (Fusion SL 3; Vilber Lourmat).

Confocal fluorescence microscopy

Immunofluorescence analysis was performed as described previously (Koh *et al*, 2017). The cells were fixed on coverslips with 4% (w/v) paraformaldehyde in PBS and then permeabilized for 10 min using 0.25% (v/v) Triton X-100 in PBS at 25 ˚C. TRAF6 or His was detected using a 1/100 dilution of the primary Ab for 1 h at 25 ˚C. After washing, the appropriate fluorescently labeled secondary Abs were incubated for 1 h at 25 ˚C. Slides were examined using laser-scanning confocal microscopy (model LSM 800; Zeiss).

MTT assay

Cell viability relative to non-treated group was measured by MTT assay, as

described previously (Kim *et al*, 2020). After incubating for the indicated time points, 5 mg/ml of MTT (3-(4,5-dimethylthiazol-2-yl)-2,5-diphenyltetrazolium bromide) solution was added in the place of media, and cells were incubated for further 4 h. Then, all the media was removed and the same volume of dimethyl sulfoxide (DMSO) solution was added for 15 min to dissolve the formazan. Using UV/VIS spectrophotometer, each well of the plate was measured at 540 nm to measure relative cell viability.

Supplementary References

Kim JS, Lee D, Kim D, Mun SJ, Cho E, Son W, Yang CS (2020) Toxoplasma gondii GRA8-derived peptide immunotherapy improves tumor targeting of colorectal cancer. *Oncotarget* 11: 62-73

Koh HJ, Kim YR, Kim JS, Yun JS, Jang K, Yang CS (2017) Toxoplasma gondii GRA7-Targeted ASC and PLD1 Promote Antibacterial Host Defense via PKCalpha. *PLoS Pathog* 13: e1006126
